# Organic Plasmon-Emitting Diodes for Detecting Refractive Index Variation

**DOI:** 10.3390/s130708340

**Published:** 2013-06-28

**Authors:** Nan-Fu Chiu, Chih-Jen Cheng, Teng-Yi Huang

**Affiliations:** Institute of Electro-Optical Science and Technology, National Taiwan Normal University, No. 88, Sec. 4, Ting-Chou Road, Taipei 11677, Taiwan; E-Mails: cjcheng1101@gmail.com (C.-J.C.); tyhuang1120@gmail.com (T.-Y.H.)

**Keywords:** surface plasmon grating coupled emission (SPGCE), directional, plasmonic band-gap, refractive-index sensor

## Abstract

A photo-excited organic layer on a metal thin film with a corrugated substrate was used to generate surface plasmon grating coupled emissions (SPGCEs). Directional emissions corresponded to the resonant condition of surface plasmon modes on the Au/air interface. In experimental comparisons of the effects of different pitch sizes on the plasmonic band-gap, the obtained SPGCEs were highly directional, with intensity increases as large as 10.38-fold. The FWHM emission spectrum was less than 70 nm. This method is easily applicable to detecting refractive index changes by using SP-coupled fluorophores in which wavelength emissions vary by viewing angle. The measurements and calculations in this study confirmed that the color wavelength of the SPGCE changed from 545.3 nm to 615.4 nm at certain viewing angles, while the concentration of contacting glucose increased from 10 to 40 wt%, which corresponded to a refractive index increase from 1.3484 to 1.3968. The organic plasmon-emitting diode exhibits a wider linearity range and a resolution of the experimental is 1.056 × 10^−3^ RIU. The sensitivity of the detection limit for naked eye of the experimental is 0.6 wt%. At a certain viewing angle, a large spectral shift is clearly distinguishable by the naked eye unaided by optoelectronic devices. These experimental results confirm the potential applications of the organic plasmon-emitting diodes in a low-cost, integrated, and disposable refractive-index sensor.

## 1. Introduction

A surface plasmon polariton (SPP) is produced by a longitudinal oscillation in surface charge density and can be resonantly excited at the metal/dielectric interface [1,2]. Recent reports indicate that emissions of an organic emitter on a metallic thin film can be enhanced by coupling SPPs via energy transfer [3,4]. These SP-coupled emissions from fluorescent molecules have potential biomedical sensing applications [5,6]. In this study of organic plasmon-emitting diodes, organic layer of tris-(8-hydroxyquinoline) aluminum (Alq_3_) emissions optically pumped by a diode laser were used to generate surface plasmon (SP) emissions by excitation of SPPs on a grating structure. The emissions correspond to the resonant condition of SP modes on the Alq_3_/Au interface and grating couple to the Au/air interface for the emission of light, which is called surface plasmon grating coupled emissions (SPGCEs) [7]. Due to local field enhancement, this phenomenon causes a viewing-angle-dependent spectral shift and an intensity modulation. This method can be readily extended to the use of SP-coupled fluorophores with different wavelength emissions to detect refractive index changes at different viewing angles. By replacing the electrically pumped organic light-emitting device (OLED) and by using a nano-grating instead of a prism, the size and price of such an SP sensor installed in disposable smart lab-on-a-chip for point-of-care testing (POCT) in clinical applications can be decreased to below those of sensors fabricated in Kretchmann configuration [7,8]. Additionally, in a conventional Kretschmann configuration, a small change in the scattered angle caused by the difference in the refractive index at the surface is detectable by monitoring a single wavelength of pumping light. In contrast, the proposed approach generates a broadband signal from the pump beam. At a certain viewing angle, a spectral shift is clearly distinguishable by the naked eye without the aid of optoelectronic devices such as a spectrometer or a photodetector [8]. Measurements of the emission band of the organic plasmon-emitting diodes revealed that such a sensor has a dynamic range (in terms of refractive index variation, Δn) as large as 0.128.

## 2. Materials and Methods

**Figure 1.**
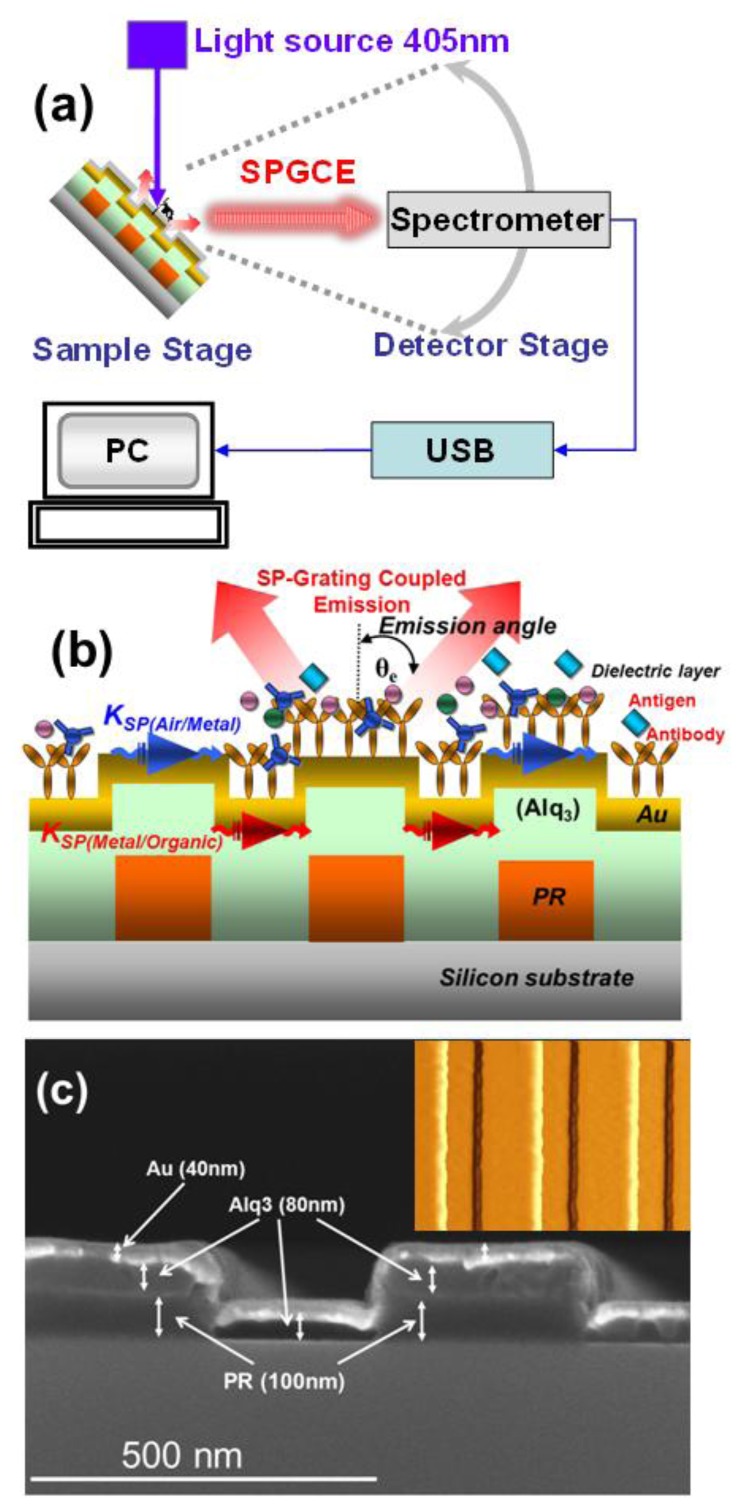
(**a**) Experimental setup of the measurement system, (**b**) schematic cross-section of the device, and (**c**) SEM and AFM (inset) images of the sample with grating pitch 500 nm.

The SPGCE-emitted light from the organic layer of Alq_3_ could couple with SPP under matching conditions while propagating along the grating surface. It can become radiative light again through the decoupling grating for directional emission. Figure 1(a) shows the experimental apparatus used to measure photoluminescence (PL) at different viewing angles. Light pumped from a 405 nm diode laser with an incident angle of 45° normal to the sample surface was used to excite the Alq_3_ with a broad spontaneous emission band (480 to 680 nm). A spectrometer mounted on the detector stage was rotated to collect the SPGCE signals scattered from the corrugated thin film structures at different viewing angles. Figure 1(b) shows the detailed cross section of the device structures. On the Si-substrate, the 400-, 500-, 600- and 800-nm photoresist (PR) 1-D gratings were first fabricated by Electron-Beam Lithography system. The high-resolution positive electron-beam photoresist (PR), ZEP520A or PMMA 950, was first spin coated on the silicon or ITO substrate to form a resist layer having thickness of 100 nm, and then pre-baked at 180 ° C for 120 s. After exposing to the electron beam of 50 kV and the current of 300 PA, nonstructural gratings having elongated one-dimensional line pattern with size of 250 nm, and pitch of 500 nm were formed. The exposure was carried out for 2 μs by a pixel map of 60,000 × 60,000 dots to give a total exposure area of 1.2 × 1.2 mm^2^ and exposure time need 1 more hour. Next, the Alq_3_ and Au layers were then deposited to obtain the corrugated structures of the Alq_3_ and Au thin films. The details of the fabrication process were reported in our previous work [7,9]. Since the dielectric materials used for biomedical applications such as *in vitro* diagnosis and drug screening are in contact with the top side of the Au layer, the spectral response of the SPGCE may differ from those shown below. Figure 1(c) and its inset show the SEM and AFM pictures of the samples with the structure of Alq_3_ (80 nm)/Au (40 nm) on the silicon substrate, respectively. The photoresist thickness is 100 nm on the silicon substrate with an exposure area of 1.2 × 1.2 mm^2^.

## 3. Results and Discussion

### 3.1. Analysis of Directional PL Emission in SPGCE Devices

**Figure 2.**
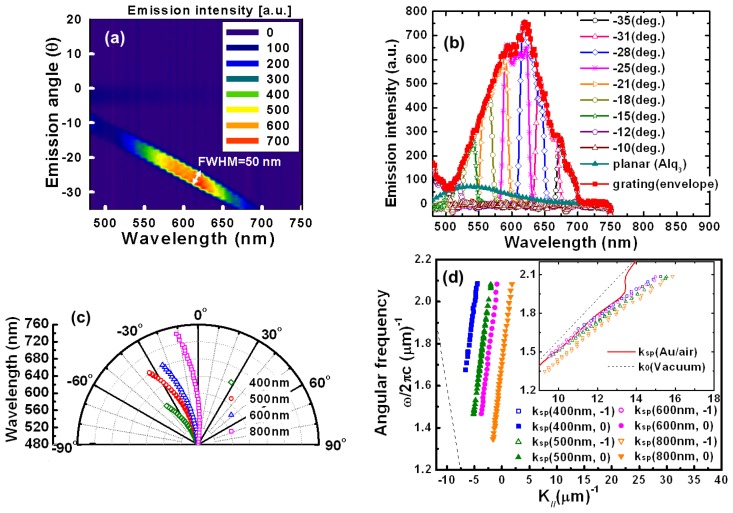
The PL emissions obtained for organic plasmon-emitting diodes (grating pitch, 500 nm; area, 1.2 × 1.2 mm). (**a**) A 3-D plot showing the PL spectra at different viewing angles of the device (grating pitch, 500 nm), (**b**) the PL of grating and non-grating (planar, Alq_3_ emission) samples as well as the integration of overall emission angles (envelope) and (**c**) spectral peaks at different viewing angles for devices with different pitches. (**d**) Relation between angular frequency and wave vector (ω-k). Solid symbols show the experimental data (m = 0) obtained at different grating pitches. Open symbols (inset) are the calculated results corresponding to m = −1. Solid and dashed lines represent calculation results for the dispersion relation at the Au/air interface and for air, respectively. The fitting results approximate the theoretical dispersion relation. The data are given for a sample with 500 nm grating pitch.

Figure 2(a) shows the 3-D plot of PL spectra at different viewing angles of the organic plasmon-emitting diode with a pitch of 500 nm. The sample has a small full-width half-maximum (FWHM) about 50 nm of the PL spectrum at different emission wavelength angles. Here, spectra were measured in increments of 1°. Figure 2(b) shows the PL spectra measurements obtained at different emission angles. Figure 2(a,b) show that emissions were strongest at −28°. At this angle, the wavelength was 620 nm, which was 10.38 fold higher than that of PL peak intensity of planar Alq_3_ (peak at 546 nm, with full width at half maximum of 100 nm). Emission angles ranging from –10° to −35° were observed in the visible spectrum from 500 to 700 nm. As the viewing angle (absolute value) increased, a red shift corresponding to a color change from greenish blue to red occurred at an angle range of −10 to −35°. These experimental results suggested that SPGCE became highly directional as light extraction efficiency increased. The enhanced PL can be due to the SP effect as mentioned above. Excitons in organic emitters transferred energy to SP mode at the metal/organic emitter interface [10,11]. Typically, a planar OLED structure cannot radiate SPs and has a quenching effect because it does not satisfy the wavevector matching condition. Introducing a corrugated structure to provide an additional in-plane wave vector satisfies the wavevector matching condition for an in-plane wave vector (*k*_//_) and a SP wave vector (*k_sp_*) in one-dimensional nanostructures. Two SPs are possible, *k_sp(Au/air)_* and, *k_sp(Au/Alq3)_*, which can be expressed as [3,4,6–8]:

(1) $$k_{//}=k_{sp(Au/Alq_{3})}=k_{Alq_{3}}\sin\theta \pm m\frac{2\pi }{\Lambda }=\frac{\omega }{c}\sqrt{\frac{\varepsilon_{Alq_{3}}\varepsilon_{m}}{\varepsilon_{Alq_{3}}+\varepsilon_{m}}}$$

(2) $$k_{0}\sin\theta \pm m\frac{2\pi }{\Lambda }=\frac{\omega }{c}\sqrt{\frac{\varepsilon_{m}}{1+\varepsilon_{m}}}$$

where *k_Alq3_* and *k_0_* are the wave vectors in organic material and air, respectively. The *θ* represents the incident angle. The *c, ε_Alq3_,* and *ε_m_* are light velocity, the relative permittivity of dielectric (organic emitters) and metal. The *m* is an integer, and **Λ** is the grating pitch [11]. The *ε_Alq3_* is wavelength-dependent and a complex value in which the real and imaginary parts are 2.979 and 0.0124, respectively, at the PL peak of the planar Alq_3_ (546 nm). Intensities of these two SPs wave (metal/air and metal/organic) were strongly dependent on device geometries and structures [10]. In our device, although there are two possible SP modes, *i.e.,* Alq_3_/Au and Au/air, only Au/air signal was observed experimentally [6,10,11]. When Au thicknesses were reduced from 40 to 20 nm, both metal/air and metal/organic SPs were observed (results not shown here). This indicates that metal/Alq_3_ SP can be impeded by Au thin film. For a biosensor application, the intensity of Au/air (Au/dielectric) mode should be increased whereas that of Au/Alq_3_ mode should be suppressed. Hence, a thicker (40 nm) Au was chosen. Figure 2(c) shows the spectral peak at different viewing angles with varying grating pitches of 400, 500, 600, and 800 nm.

The spectra clearly show the highly directional coupling emissions. As the grating pitch decreases, the grating diffraction term (2π/Λ) in Equations (1) and (2) increases, which then increases the diffraction angle (in absolute value). Figure 2(d) shows how, based on the measurements for different pitch samples, Equation (2) was used to calculate the peak emission wavelength at each emission angle based on its dispersion curve (ω-k relation). Closed symbols represent the measured ω-k relations extracted from Figure 2(c), which fall within the light cone defined by the two dashed lines in Figure 2(d). Therefore, the wavevector matching conditions obtain an efficient emission to the air in SP mode, which corresponds to the case of m = −1 in Equation (2). Open symbols show the calculation results obtained when the momentum terms (2π/Λ) corresponding to m = 0 were added for different pitches. In this case, the ω-k relation is independent of the grating pitch. With the decreasing grating pitch, grating diffraction term (2π/Λ) in Equation (2) increases, which results in a larger shift in momentum space from m = 0. Although some grating periods (e.g., 600 and 800 nm) were larger than those of the Alq_3_ emission band (480-680 nm), grating applied according to Equation (2) provided the additional in-plane wavevector needed to satisfy the wavevector matching condition.

Table 1 summarizes the range of emission angles (*θ_e_*) and peak emission angles (MP *θ_e_*) obtained for air and water of SPGCE when the pitch was experimentally varied. The calculation results for momentum shift (ΔK) are also shown.

**Figure 3.**
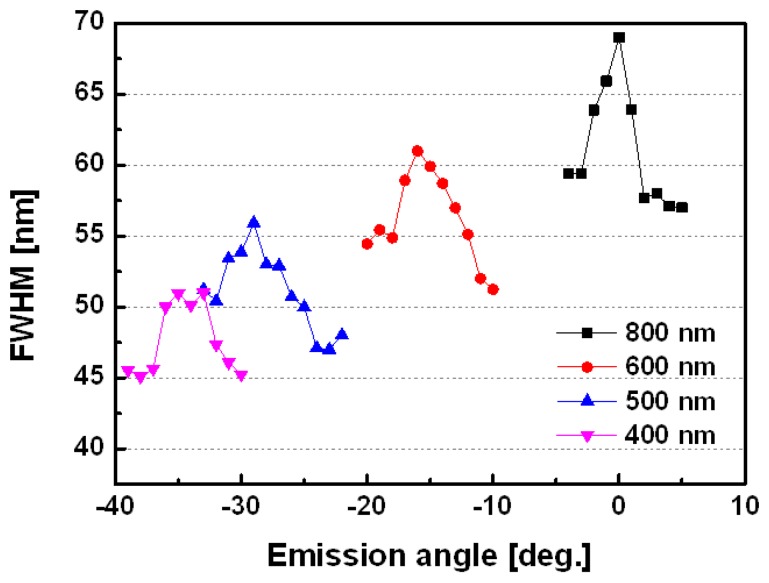
The experimental results show that the measurement of FWHM *vs.* emission angles for pitch size of 400 nm to 800 nm devices.

Figure 3 shows the four organic plasmon-emitting diodes for full-width half-maximum (FWHM) ranges from 45 to 70 nm of the PL spectrum at different emission wavelength angles. The 400 nm structure had an FWHM range of 45-53 nm, which is narrower than that of the 800 nm structure, which had a range of 57-70 nm, probably due to the stronger intensity of the 400 nm devices.

### 3.2. SPGCE for Sensor Applications

**Figure 4.**
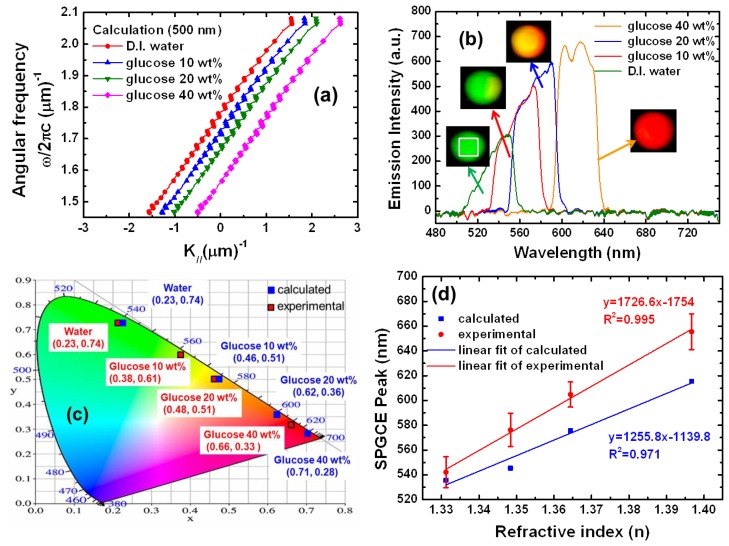
Experimental and calculation results for pitch size of 500 nm. (**a**) Dispersion relation calculated for different species contacting the top side of the sample; (**b**) experimental results showing the SPGCE sensor at difference dielectrics of the sample; (**c**) shift in experimental and calculated CIE coordinates for different species at a fixed viewing angle; and (**d**) the calibration curve for the determination of D.I. water and in 10 wt%, 20 wt%, and 40 wt% glucose solutions for the sample.

For sensor applications, materials applied to the Au layer have different refractive indices if they have different dielectric constants (*ε_d_*). Here, Equation (2) should be modified as follows [8,9]:

(3) $$\frac{\omega }{c}\sqrt{\frac{\varepsilon_{d}\varepsilon_{m}}{\varepsilon_{d}+\varepsilon_{m}}}=k_{d}\sin\theta \pm m\frac{2\pi }{\Lambda }$$

where *k_d_* is the wave vector and *ε_d_* is the dielectric of the sample assay. An *ε_d_* value of 1 corresponds to air and converges to Equation (2). However, realizing such a device (dielectric on thin Au) is difficult in this experimental stage. A dielectric (such as solution) that contacts the thin metal film easily penetrates the Au, attacks the Alq_3_, and degrades the PL intensity. Therefore, a suitable passivation thin film is needed to protect the devices [12,13]. According to the results of the experiment and calculations, the grating pitch was set to 500 nm. The *ε_m_* values of Au at different wavelengths are given in [14]. Here, deionized water (D.I. water) has an *ε_d_* of 1.772, and glucose with 10%, 20%, and 40% (weight percentage) have *ε_d_* values of 1.818, 1.862, and 1.951, respectively. In SPGCE sensors with angular interrogation mod (angular modulation e), a fixed wavelength (angular frequency) of emitted angle *(K_//_* is the wave vectors parallel to the surface of the emitted light) is used to excite an SPGCE (Figure 4(a)). The propagation constant of the SPP and its changes are determined by measuring the intensity of emitted light at different angles of determining the angle of emission yielding the strongest coupling with an SPP occurs. Figure 4(a) shows the calculation results obtained by Equation (3) for the above parameters. As *ε_d_* increases, a right shift of the ω-k curves occurs, which corresponds to an increase in momentum space.

In SPGCE sensors with wavelength modulation (wavelength interrogation mode), a fixed angle *(K_//_)* of emitted light (angular frequency) is used to excite an SPGCE (Figure 4(a)). The propagation constant of the SPP and its changes are determined by measuring the intensity of emitted light at different wavelengths and determining the color gradient at which the strongest coupling with an SPP occurs (Figure 4(b)). Figure 4(b) shows the experimental results obtained at fixed emission angle of −17° for different solutions pumped into the flow cell of the D.I. water and the glucose solutions.

The SPGCE devices were embedded in solutions containing D.I. water and 10 wt%, 20 wt% and 40 wt% glucose solutions. The solutions were sequentially injected at a flow rate of 400 uL/min into the device pre-filled with glucose. The entire volume of the flow cell (1 mL) was exchanged over a 3 min period. After each injection, the D.I water was washed with the different glucose concentrations at the same flow rate. The four emission spectra obtained for D.I. water and for 10 wt%, 20 wt% and 40 wt% glucose were green, which was also the color of the surrounding glucose solution. When the surrounding glucose solution was varied, however, the color changed from green, to orange, and to red, depending on the glucose concentration. Figure 4(c) shows the CIE 1931 color space chromaticity diagram with the calculated color point for the spectrum shown in Figure 4(b). To visualize the color shift, Figure 4(c) plots the chromaticities for the experimental and calculation results in the CIE color space. At the calculation data, for water, the CIE color was 542.13 nm (0.23, 0.74). For 10 wt%, 20 wt%, and 40 wt% glucose solutions, the CIE colors were 576.28 nm (0.46, 0.51), 604.83 nm (0.62, 0.36), and 655.57 nm (0.71, 0.28), respectively. At the experimental results, for water, the CIE color was 535.5 nm (0.23, 0.74). For 10 wt%, 20 wt%, and 40 wt% glucose solutions, the CIE colors were 545.3 nm (0.38, 0.61), 575.6 nm (0.48, 0.51), and 615.4 nm (0.66, 0.33), respectively. This experiment further confirmed that these colors are clearly distinguishable by the naked eye. Figure 4(d) shows that the calculated and experimental determined SPGCE characteristics in wavelength interrogation mode exhibited a good agreement with the calibration curve. The experimentally result of sample gave a linear plot for the range of water to 40 wt% glucose, with a linear regression equation y = 1726.6x − 1754 and a correlation coefficient (R^2^) of 0.995, where y represents the wavelength of emission peak and x correspond the refractive index (n). The performance of SPGCE biosensors is evaluated in terms of its resolution and sensitivity, as shown in Table 2. The results show that the resolution calculated of this organic plasmon-emitting diode using the wavelength interrogation mode is shown in Equation (4) [15]:

(4) $$\sigma_{n}=\frac{\Delta n}{\Delta \lambda }\sigma_{\lambda }$$

where *σ_n_* is the resolution in terms of RIU. The Δ*n* and Δ**λ** are changes in refractive index and corresponding wavelengths, respectively. The *σ_λ_* is the wavelength resolution of the spectrometer. For glucose concentrations of 20 wt% and 10 wt%, the refractive index was adjusted from 1.3644 to 1.3484, respectively, which corresponds to Δn = 0.016 RIU.

Figure 4(b) shows the experimental measurements based on *δn* and *δλ* values for glucose concentrations of 20 wt% and 10 wt% for a wavelength shift from 575.6 to 545.3 nm at a measurement angle of 1°. The resolution of the measurement system is:

(5) $$\sigma_{n}=\frac{\Delta n}{\Delta \lambda }\sigma_{\lambda }=\frac{(1.3644-1.3484)\text{RIU}}{(575.6-545.3)nm}\times 0.3nm=1.58\times 10^{-4}\;\text{RIU},\;\text{for experimental}$$

(6) $$\sigma_{n}=\frac{\Delta n}{\Delta \lambda }\sigma_{\lambda }=\frac{(1.3644-1.3484)\text{RIU}}{(604.83-576.28)nm}\times 0.3nm=1.68\times 10^{-4}\;\text{RIU},\;\text{for calculated}.$$

This RIU value is not as high as that obtained by other SPR sensors to detect small molecules (e.g., 5 × 10^−7^ RIU) [15-17]. However, the value is maybe sufficient for a biosensor designed to detect large molecules with micron-size (2∼4 μm) such as those used to diagnose *Mycobacterium tuberculosis* (MTB) cells in blood serum [18].

The specific binding technique used on the biosensor surface requires a 2.2 × 10-^4^ change in the refractive index [19]. Note that the spectrometer used in this setup has a resolution of 0.3 nm, but its small physical size makes it highly portable, as shown in Table 2. Use of a high resolution spectrometer (Δ*λ* = 0.01) improved sensitivity to 5.28 × 10^−6^ [20].

Figure 4(c) is a chromaticity diagram showing the CIE coordinates for different dielectric materials observed at −13°. Notably, as *ε_d_* increases the color varies from green (D.I. water), to yellow (glucose 10 wt%), to orange (glucose 20 wt%), and to red (glucose 40 wt%). The device emits light with different ranges of visible spectra when it contacts species with different relative permittivities. Therefore, at certain viewing angles, signal detection can be performed by the naked eye and without a spectrometer. Attaching an optical film to limit the viewing angle provides a device appropriate for disposable and point-of-care biosensors. Since the human eye can discriminate a wavelength approximating 2 nm [21], resolution is:

(7) $$\sigma_{n}=\frac{\Delta n}{\Delta \lambda }\sigma_{\lambda }=\frac{(1.3644-1.3484)\text{RIU}}{(575.6-545.3)nm}\times 2nm=1.056\times 10^{-3}\;\text{RIU},\;\text{for}\;\text{experimental}$$

(8) $$\sigma_{n}=\frac{\Delta n}{\Delta \lambda }\sigma_{\lambda }=\frac{(1.3644-1.3484)\text{RIU}}{(604.83-576.28)nm}\times 2nm=1.121\times 10^{-3}\;\text{RIU},\;\text{for calculared}.$$

Since this RIU value is quite low, the naked eye may be unsuitable for detecting the change in refractive index in a thin film such as that used in hybridization-adsorption biosensors, which have high specificity with small refractive index change [22]. A more appropriate use would be for detecting refractive index change in liquid, such as determining protein and glucose concentrations in urine and serum, and fat concentrations in milk and hydroxyl content in soybean oil, with Δ*n* at 10-^3^ range [23-25], as shown in Table 2.

Two advantages of the sensor are simple system configuration and its wide dynamic range. Compared with conventional Kretschmann configuration, the function of the light source is to pump the organic emitter. Hence, careful optical alignment is unnecessary. Using electroluminescence instead of an optical pump completely eliminates the need for a light source in the system, which further simplifies the system. Additionally, the sensor has a wide dynamic range in terms of refractive index variation. Since the visible range is only limited by the emission band of the organic emitter, it can be as large as 0.128. Extending multiple emitters into the infrared region can further increase dynamic range [26,27].

## 4. Conclusions

This study developed a highly directional organic plasmon-emitting diode in which grating pitches are adjusted according to Au/air SP radiation and with an FWHM lower than 70 nm. Based on a simple calculation, the ω-k relation fit well with the experimental results, which were extracted from the spectral peak of the viewing angle dependent spectra. Experiments showed that the strong coupling photonic resonance in SPP grating resonances in Au/air was 10.38 times higher than that of PL peak intensity of planar Alq_3_. When in contact with various materials, SPGCE showed high sensitivity to the dielectric constant. This experiment showed that, with the naked eye, the SPGCE method can detect the change of refractive index as small as 1.056 × 10^−3^. At certain viewing angles, the broadband emissions from the organic material under varying *ε_d_* reveal clear color changes. Potential applications include low-cost diagnostic tools and disposable sensors.

## Figures and Tables

**Table 1.** Experimental results showing the SPGCE emission angle (*θ_e_*), emission angle with maximum emission intensity (MP*θ_e_*), and momentum shift (ΔK) at different pitches.

Pitch (nm)	*θ_e_*	ΔK(*μ*m)^−1^	Order (m)	MP*θ_e_*	

				Air	Water
400	−40° ∼ −30°	18.98234	−1	−37°	−25°
500	−35° ∼ −10°	16.74774	−1	−28°	−17°
600	−25° ∼ −5°	14.43099	−1	−15°	−5°
800	−10° ∼ 10°	13.52638	−1	0°	8°

*θ_e_*, emission angle. MP*θ_e_*, maximum peak emission angle.

**Table 2.** Performance of the resolution for the organic plasmon-emitting diode.

SPGCE Pitch Size 500 nm	Resolution (RIU)	

	Experimental	Calculated
Detector for spectrometer (0.3 nm)	1.58 × 10^−4^	1.68 × 10^−4^
Detector for naked eye (2 nm)	1.056 × 10^−3^	1.121 × 10^−3^
Slope	1726.6	1255.8
Detection limit for spectrometer	0.09 wt%	0.3 wt%
Detection limit for naked eye	0.6 wt%	2 wt%

## References

[1] Wood R.W. (1902). On a remarkable case of uneven distribution of light in a diffraction grating spectrum. Phil. Mag..

[2] Raether H. (1988). Surface Plasmons on Smooth and Rough Surface and on Gratings.

[3] Gifford D., Hall D.G. (2002). Extraordinary transmission of organic photoluminescence through an otherwise opaque metal layer via surface plasmon cross coupling. Appl. Phys. Lett..

[4] Feng J., Okamoto T., Kawata S. (2005). Highly directional emission via coupled surface-plasmon tunneling from electroluminescence in organic light-emitting devices. Appl. Phys. Lett..

[5] Lakowicz J.R., Malicka J., Gryczynski I., Gryczynski Z. (2003). Directional surface plasmon-coupled emission: A new method for high sensitivity detection. Biochem. Biophys. Res. Commun..

[6] Winter G., Barnes W.L. (2006). Emission of light through thin silver films via near-field coupling to surface plasmon polaritons. Appl. Phys. Lett..

[7] Chiu N.-F., Yu C., Nien S.-Y., Lee J.-H., Kuan C.-H., Wu K.-C., Lee C.-K., Lin C.-W. (2007). Enhancement and tunability of active plasmonic by multilayer grating coupled emission. Opt. Exp..

[8] Homola J., Yee S.S., Gauglitz G. (1999). Surface plasmon resonance sensors: Review. Sens. Actuators B Chem..

[9] Chiu N.-F., Lin C.-W., Lee J.-H., Kuan C.-H., Wu K.-C., Lee C.-K. (2007). Enhanced luminescence of organic/metal nanostructure for grating coupler active long-range surface plasmonic device. Appl. Phys. Lett..

[10] Wedge S., Giannattasio A., Barnes W.L. (2007). Surface plasmon-polariton mediated emission of light from top-emitting organic light-emitting diode type structures. Org. Electron..

[11] Nien S.-Y., Chiu N.-F., Ho Y.-H., Lee J.-H., Lin C.-W., Wu K.-C., Lee C.-K., Lin J.-R., Wei M.-K., Chiu T.-L. (2009). Directional photoluminescence enhancement of organic emitters via surface plasmon coupling. Appl. Phys. Lett..

[12] Burrows P.E., Bulovic V., Forrest S.R., Sapochak L.S., McCarty D.M., Thompson M.E. (1994). Reliability and degradation of organic light emitting devices. Appl. Phys. Lett..

[13] Yun S.J., Ko Y.W., Lim J.W. (2004). Passivation of organic light-emitting diodes with aluminum oxide thin films grown by plasma-enhanced atomic layer deposition. Appl. Phys. Lett..

[14] Smith D.Y., Shiles E., Inokuti M., Palik E.D. (1985). Handbook of Optical Constants of Solids.

[15] Nelson S.G., Johnston K.S., Yee S.S. (1996). High sensitivity surface Plasmon resonance sensor based on phase detection. Sens. Actuators B Chem..

[16] Chang C.-C., Chiu N.-F., Lin D.S., Chu S.-Y., Lin C.-W. (2010). High-sensitivity detection of carbohydrate antigen 15-3 using a gold/zinc oxide thin films surface plasmon resonance-based biosensor. Anal. Chem..

[17] Mitchell J. (2010). Small molecule immunosensing using surface plasmon resonance. Sensors.

[18] Yeo W.-H., Chou F.-L., Fotouhi G., Oh K., Stevens B.T., Tseng H.-Y., Gao D., Shen A.Q., Chung J.-H., Lee K.-H. (2010). Size-selective immunofluorescence of *Mycobacterium tuberculosis* cells by capillary- and viscous forces. Lab. Chip.

[19] Nagel T., Ehrentreich-Förster E., Singh M., Schmitt K., Brandenburg A., Berka A., Bier F.F. (2008). Direct detection of tuberculosis infection in blood serum using three optical label-free approaches. Sens. Actuators B Chem..

[20] Akimoto T., Sasaki S., Ikebukuro K., Karube I. (2000). Effect of incident angle of light on sensitivity and detection limit for layers of antibody with surface plasmon resonance spectroscopy. Biosens. Bioelectron..

[21] Wyszecki G., Stiles W.S. (1982). Color Science.

[22] Jordan C.E., Frutos A.G., Thiel A.J., Corn R.M. (1997). Surface plasmon resonance imaging measurements of DNA hybridization adsorption and streptavidin/DNA multilayer formation at chemically modified gold surfaces. Anal. Chem..

[23] Liu Y., Hering P., Scully M.O. (1992). An integrated optical sensor for measuring glucose concentration. Appl. Phys. B.

[24] Jääskeläinen A.J., Peiponen K.E., Räty J.A. (2001). On reflectometric measurement of a refractive index of milk. J. Dairy Sci..

[25] Xie W., Li H. (2006). Hydroxyl content and refractive index determinations on transesterified soybean oil. J. Am. Oil Chem. Soc..

[26] Hsiao C.-H., Lee J.-H. (2009). Emitting-layer design of white organic light-emitting devices with single-host material. J. Appl. Phys..

[27] Choudhury K.R., Song D.W., So F. (2010). Efficient solution-processed hybrid polymer–nanocrystal near infrared light-emitting devices. Org. Electron..

